# Skin Microbial Changes during Space Flights: A Systematic Review

**DOI:** 10.3390/life12101498

**Published:** 2022-09-26

**Authors:** Pamela Tozzo, Arianna Delicati, Luciana Caenazzo

**Affiliations:** Legal Medicine Unit, Department of Cardiac, Thoracic, Vascular Sciences and Public Health, University of Padova, 35121 Padova, Italy

**Keywords:** skin microbiome, skin microbiota, space flight, International Space Station, dermatology, aerospace medicine

## Abstract

**Background**. Sixty years after the launch of the first human into space, different studies on the physiological changes that humans undergo during dynamic flight phases and prolonged weightlessness have been undertaken. Understanding these changes is important for the creation of the preventative measures that are essential to ensuring astronaut health. Among these changes, those of the skin are frequent, despite being rarely treated during missions. The skin is a physical barrier that protects the body from pathogen invasion and environmental changes, and it harbors diverse microbial communities that form the skin microbiota. **Methods**. A systematic literature review of skin microbiome changes during space flight was conducted using public electronic databases (PubMed and Scopus) selecting studies published from 2015 to 2022. The systematic review was performed according to 2020 PRISMA guidelines. **Results**. A total of 17 studies were collected and, after screening for inclusion and exclusion criteria, eight studies were included in this review. According to the examined literature, some skin microbiota changes seems to be only temporary, in particular *Gamma*- and *Betaproteobacteria* abundance tends to decrease, while the occurrence of the *Malassezia* species and Firmicutes, including *Staphylococcus* and *Streptococcus*, tends to increase. At the same time, there seems to be an exchange of microorganisms between astronauts and between the confined environment and a single astronaut, with alterations in the proportion of microorganisms maintained during the flight, in particular for species such as *Corynebacterium* spp., *Staphylococcus* spp., *Streptococcus* spp. and *Cloacibacterium* spp. Given that skin contributes both to protecting the body from pathogen invasion and environmental changes and to maintaining human homeostasis, changes in the skin microbiota of astronauts might result in skin diseases. **Discussion**. The skin microbiota of astronauts seems to influence the microbial composition of the International Space Station, but further studies should be performed to better understand skin microbiota dynamics and to prevent the development of dermatologic conditions during space flight.

## 1. Introduction

Since the launch of Russian cosmonaut Yuri Gagarin into orbit in April 1961, the physiological changes that the human body undergoes during dynamic flight phases and prolonged weightlessness have been widely studied. Understanding the abovementioned changes that occur as the human body adapts to the space flight environment is important for developing countermeasures and protections against the common medical problems encountered and their deleterious effects [1]. “Space medicine” is a clinical discipline that studies astronaut health; in particular, it deals with pre-mission screening to prevent diseases, healthcare delivery during missions, and long-term recovery and restoration of post-mission astronaut health status [2]. The fact that space flight may increase the risk of isolation from medical experts means that preventative measures are essential to ensuring astronaut health and to optimizing human performance; the ultimate goal of this specialty is to promote the safety of humans during exposure to the stresses of aerospace flight, such as extreme temperatures, low atmospheric pressure, radiation, noise, vibration, lack of oxygen, and strong acceleration and deceleration forces. Other risks of space flight include weightlessness, motion sickness, pilot fatigue, discomfort due to hunger or sleepiness due to the absence of the Earth’s day and night cycle, and psychological disturbances caused by confinement and isolation. These problems, however, are generally prevented by intensive preflight training in high-powered simulators and the careful design of equipment and spacecraft [3]. Even if the astronaut’s life and/or the mission are rarely threated by dermatologic conditions, since dermatologic complaints are among the most commonly reported medical events during space flight, the manifestation and the management of dermatologic events have to be taken into account while planning a space flight [4].

The International Space Station (ISS) is the largest object in the Earth’s orbit located approximately 400 km above the Earth. The ISS constitutes the most important and ambitious global cooperation program undertaken in the scientific and technological field to date, and can be considered the greatest engineering work ever created by man. The ISS is a large multinational scientific laboratory, where many experiments are conducted in a wide range of disciplines including: physics, biotechnology, chemistry, meteorology, materials science and astronomy. The ISS mainly studies how things behave in conditions of microgravity and the effects of cosmic radiation on astronauts. It is a confined and closed habitat with unique conditions such as cosmic radiation and microgravity, which induce skin changes. Furthermore, during space flight, it is difficult to perform the everyday activities that are easily performed on Earth, such as taking a shower. Therefore, during their stay in the ISS, astronauts keep clean mostly by using wet tissues and washing their hair with rinse-less shampoos, and they cannot wash their clothes or change them as often as they do on Earth [5,6].

Some of the skin physiologic changes following a six-month mission described to date include: an increase in dermal collagen production, up to 143% of normal production; epidermal thinning, with the epidermal thickness reduced to 30 micrometers compared with normal epidermis thickness, which ranges from 27 to 150 micrometers; and a loss of dermal elasticity [7,8,9,10,11].

Human skin is composed of two main layers: the epidermis and the dermis. As the largest organ of the human body, the skin physically separates, but metabolically connects, the outside environment and the internal tissue. The skin can protect the body from pathogen invasion and environmental changes, and influences the human body’s homeostasis. The human body harbors trillions of microbes that form a diverse ecosystem, including bacteria, virus, fungi and protozoa; collectively, they are named “human microbiota” and their genomes are referred as the “human microbiome” [12,13]. Bacteria are distributed differently in the human body: we can find them in the gastrointestinal tract (70%), in the skin (not only in the outermost layers, but also in the deeper ones), in the vaginal mucosa and in the respiratory tract; in addition, more recently, they have also been found in other areas of the body such as the mammary gland and the bladder [14].

The most consistent colonization occurs in the gastrointestinal tract, where it is possible to find approximately 70% of the resident microbial community, mainly represented by anaerobic bacteria such as Proteobacteria and Actinobacteria. This is the district most subject to evolution over time before the definitive adult, stable microbial composition is reached, which is why neonatal periods and childhood are important phases in the constitution of the intestinal microbial community and its influence on the development and maturation of the immune system [15]. 

It has already been shown that the particular composition of human microbial communities is influenced by many factors, such as body location [16], diet [17], sex [18,19] and age [20,21]. The predominant bacterial phyla in the human microbiome are: Actinobacteria, Firmicutes, Proteobacteria, Bacteroidetes, Cyanobacteria and Fusobacteria, but the predominance of one phyla over another varies among individuals. Furthermore, if a single individual is taken into consideration, it is possible, even within that individual, to identify a different distribution depending on the body site that is analyzed. In particular, a microbial community can also be shaped by habits [22], relationships [23], disease state [24] and environment [25]. For centuries, microbes have been seen as a threat to human survival, so recent discoveries regarding the close collaboration with and importance of microbes for the human body have been a real revelation. These close symbiotic alliances were created following various processes of coevolution. If you consider that each of us comprises 40 trillion human cells and 100 trillion microbial cells, it is easy to understand the importance of these microorganisms in our biological system, carrying out both metabolic and physiological functions, and above all, how an alteration in the microbiota (dysbiosis) can be the cause of major diseases. Therefore, understanding the changes in a specific microbiome and metabolic profile in some body sites (for example, gut or saliva) in steady long-term isolation confinement can help to establish the change rule of the human microbiome and the possible disease risks in long-time space travel [26].

The skin microbiota consists of bacteria, eukaryotes and viruses. Skin diseases may develop if the fine balance among skin microbial communities is disrupted by microorganism or host factors. As with the intestinal microbiota, skin microbiota also undergoes a progressive formation process, from life in utero to the first years of life [27]. Already during gestation, the fetus comes into contact with amniotic fluid and a first transfer of microbes takes place. The actual transfer, however, occurs at the time of birth, when the fetus passes through the mother’s vaginal canal. This first microbial flora is mainly represented by maternal vaginal microbes or microbes taken from the external environment. This first colonization is of fundamental importance because, in a very short period of time, there is an influx of highly activated and subsequently inhibited regulatory T cells, so that the microbial flora begins to create a symbiotic relationship with the body’s immune system. This will be the initial step for the formation of the skin microbiota that will develop simultaneously with the immune system. The maturation of the skin microbiota will continue until 12–18 months of life, when it will reach a composition similar to that of the adult. The infantile *stratum corneum*, in fact, is more hydrated than that of the adult, so there will initially be a predominance of staphylococci. During the first year of life, then, the humid areas will tend to decrease and the baby’s skin will become more and more similar to that of the adult, becoming site-specific and presenting a greater microbial component represented by the phyla Firmicutes. Skin changes after birth mainly include changes in pH, trans-epidermal water loss and sebum production; however, the development of the barrier function of the skin, the keratinization process and the development of the immune system are all factors that can also influence the correct bacterial colonization. The consequence of this process can be either a correct development and therefore an intact microbiota, with adequate microbial richness and diversity capable of resisting colonization by pathogenic microbes, or, otherwise, an absence of maturity and stability. Furthermore, the microbiota plays a crucial role in the development of the immune system, and a dysbiosis can lead to the development of subsequent diseases such as atopy or hyperactivity [28]. Dysbiosis has been described as an imbalance between the microbiota and its host, or as a form of altered homeostasis in which the microbiota is shifted to a less complex pathological spectrum. Today, they are extensively studied in an attempt to understand and identify the precise causes of the alteration and the phenomena that occur, leading to the development of new targeted therapies [29].

In recent years, with the development of space medicine, shifts in the skin microbiome composition of astronauts have also been documented. Furthermore, the fact that every hour, a single human occupant can release approximately 10 million bacteria or fungi inside a built environment [30] has raised concerns about astronauts’ health. In fact, the astronauts that are supposed to live for long periods inside the closed environment of the ISS can become more susceptible to infection during long space flight missions due to a weakening of the immune system, as shown by the reactivation of the Epstein–Barr virus (EBV) and the varicella-zoster virus (VZV) during space flight [31,32]. Moreover, it should be considered that cutaneous alterations represent a major concern for astronauts, since different cutaneous signs and symptoms have been described during and after space flights, such as erythema, peeling, dryness, burning, pruritus, sensitivity, thinning and delayed wound healing [6].

From the studies published in recent years, there is evidence that bacteria can survive the environmental conditions of space, and this data also opens up new research opportunities regarding the adaptation of man to space. In fact, microgravity determines adaptations at the genotypic and phenotypic level that can favor the growth, as well as the virulence, of some bacterial species. As a result, the human microbiome can undergo changes when exposed to microgravity and radiation [33]. It is important to underline that, historically, microbiology was almost entirely culture-dependent. Therefore, early studies of the human microbiome involved the culturing of microbes. Since many skin bacteria are difficult to grow, prior to the advent of next-generation sequencing (NGS) technologies, only a limited set of information about the skin microbiome was available. Furthermore, microorganisms sharing similar morphologies and functional characteristics were difficult to distinguish using culture-dependent methods. With the advent of culture-independent methods, it became possible to reach a deeper lever of microbial identification. What is of paramount importance for astronauts’ health is to maintain the balance of skin microbiota to avoid skin diseases and to prevent skin infections. This review aims to present and evaluate the current knowledge regarding the skin microbiota changes that the human body undergoes during dynamic flight phases and prolonged weightlessness.

## 2. Materials and Methods

This review was performed in accordance with the Preferred Reporting Items for Systematic Reviews and Meta-Analyses and the PRISMA Guidelines [34]. 

A systematic literature review regarding skin microbiome changes during space flight was conducted using public electronic databases (PubMed and Scopus) published from 2015 to 2022. The works were selected according to the query: “((microbiota) OR (microbiome)) AND ((astronaut) OR (space station)) AND (skin)”. Results management was performed with the use of Microsoft Office software such as Excel and Word. Zotero software was used to edit and organize the bibliography.

The exclusion criteria were: (1) not relevant publication (not related to the topic); (2) reviews; (3) letters or editorials; (4) articles in a language other than English; and (5) animal studies. The inclusion criteria were: (1) original article; (2) article in English full text; and (3) studies performed on humans.

A total of 17 works were identified through the database search. An English language filter was applied to start the screening process, narrowing the search to 16 works. No duplicates were found. The process then continued through the screening of titles and abstracts, followed by the evaluation of the full text of those works not excluded on the basis of the latter. All 16 works were thus examined on the basis of title and abstract, after which the full texts of 10 articles were examined for eligibility. In this last phase of selection, 2 articles were excluded. Therefore, a total of 8 articles published from 2015 to 2022 were selected for qualitative synthesis.

**Figure 1:**
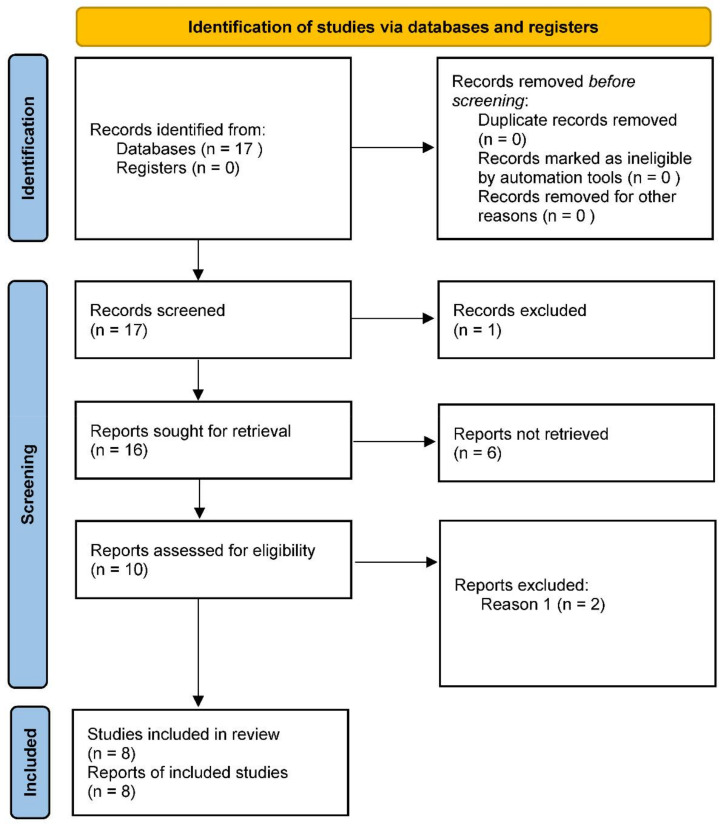
PRISMA 2020 flow diagram for new systematic reviews that included searches of databases and registers only.

The number of articles excluded or included were registered and reported in the PRISMA flowchart (Figure 1). This review was not registered and there is no specific protocol for this review.

The main findings of the 8 studies included in this review are summarized in Table 1.

## 3. Results and Discussion

In the twentieth century, all microbial communities were able to be studied using in vitro cultivation, but it remained difficult to reproduce the specific microenvironments in which microbial species could be isolated. Subsequently, the development of new methods of analysis has allowed the launch of many research projects into the instrument of the microbiome, which aim not only to collect taxonomic and functional information, but also to understand the interaction between microorganism communities and the human body, their influence on digestive and metabolic systems, and on human development and physiology. In recent years, the rapid advances in molecular sequencing and computational techniques have greatly improved the amount of sequencing data that is available for microbial analysis. The most used method is that of sequencing the 16S rRNA subunit. As is well known, this is an essential component of the small unit of ribosomes containing a specific sequence for each bacterial species, and it is therefore used for the analysis of the composition of microbial communities. The 16S rRNA is in fact sequenced through NGS platforms, and similar sequences are grouped into OTUs (operational taxonomic units), which represent a way to distinguish species and classify nucleotide sequences in different taxonomic levels. The abundance of different OTUs is then estimated on the basis of the number of corresponding sequences. A different process instead is a metagenomic approach, in which the entire genome collected from the microbiota is sequenced, that is, from all the microorganisms present in a given site, often resorting to the “shotgun” alternative; in this case, the genome is first randomly fragmented, proceeding with the amplification and sequencing of the individual pieces. The reads obtained can be assembled to form longer sequences called “contigs”, to reconstruct the order of the original DNA chains, prior to fragmentation. The assembly of the reads takes place thanks to special software that identifies the portions of the sequence that they have in common.

The studies included in this literature review show that interest in the effect of space flight on skin physiology and pathophysiology has been growing over the past 15 years. In particular, the studies had different objectives; for example, to establish the temporal changes in skin microbiota induced by the astronauts staying in space stations, to analyze the possible correlation between these changes and some structural or pathological alterations in the skin, and to understand the mechanisms underlying microbial transfer, in particular among crew members and between the confined built environment and the astronauts. The importance of these studies derives, above all, from the fact that the precise mechanisms of interaction between the skin and the microorganisms in the mucosal immune response and the variations in proportions between the various genera and species, both in physiological conditions and in various skin pathologies, are not yet fully known.

The first important point to note is that during the stay in space, it seems that skin colonization by the *Malassezia* species increases, and then decreases after the return of the astronauts to Earth, probably due to the methods with which skin hygiene is implemented during the flight. Indeed, in 2010, using pyrosequencing of the D2 LSU rRNA gene and quantitative PCR assay, Sugita et al. [36] analyzed the temporal changes in the skin fungal microbiota of 10 astronauts before, during and after their stay in the ISS (mean duration of stay was 5.5 months) by collecting a total of four samples for each astronaut (cheek and chest samples): once prior to the astronauts’ trip to the ISS, twice during their stay at the ISS (at 2 and 4 months) and once after their return to Earth. The authors noticed that the predominant fungi were the lipophilic skin fungi belonging to nine *Malassezia* species, which were identified on most samples, regardless of the collection period, body site (cheek or chest) or subject. During their stay in the ISS, the fungal diversity was reduced, and the ratio of *Malassezia* spp. to all fungal colonization increased, while colonization by *Malassezia* spp decreased on the astronauts’ return to Earth. The authors linked the increased percentage of *Malassezia* spp. to stress or to the body-washing method necessitated by space flight, in which astronauts dry-wash their body and hair. The *Malassezia* species are responsible for the development of seborrheic dermatitis, suggesting that astronauts are more likely to develop this dermatological disease during space flight. Even if reduced during the space flight, fungal diversity was recovered post-flight. One interesting finding was that the ascomycetous yeast *Cyberlindnera jadinii*, an environmental fungus, was abundantly detected in the in-flight skin samples from 5 of the 10 astronauts. Since the yeast was already detected in 3 pre-flight skin samples from these 5 astronauts, the authors inferred that the microorganism may have incidentally adhered to the skin during the pre-flight period and persisted on the skin thereafter, highlighting that the incidental entry of unexpected microorganisms into the station might jeopardize the health of astronauts and should therefore be investigated and taken into account when programming the in-flight management of astronauts’ health. Even if, in this study, none of the astronauts showed particular skin problems during the mission, the merit of this study is to have underlined the temporal variability of the fungal microbiota during the stay in the ISS, suggesting the importance of monitoring the temporal variability of microbiota even for longer space missions (more than 5 or 6 months). 

Before this study, Sugita et al. [35] had already suggested that the *Malassezia* species, as a representative lipophilic skin microorganism, could be used as a microbiological marker of the hygienic condition of skin in pseudo-space environments, where the ability to bathe is restricted. They investigated temporal changes in the level of colonization by the *Malassezia* species by comparing skin samples (scalp, cheeks, anterior chest, ear and soles of the feet) from geological investigation team members collected before, during and after their trip to Antarctica, where they were subjected to “pseudo-space” environment conditions, including the inability to bathe or shower. The choice to study the members of two geological Japanese Antarctic Research Expeditions in Antarctica derives from the fact that the anomalous duration of the hours of sunshine and the forced coexistence of researchers in closed environments make the conditions in Antarctica very similar to life in the International Space Station. Not being able to access frequent and traditional washing practices, such as taking a shower, the level of sebum on the skin of the researchers increased, favoring an increase in colonization by lipophilic microorganisms. Therefore, the authors showed a temporal increased level of *Malassezia* species colonization, irrespective of body site, which returned to the level that it was before the trip to Antarctica once they had returned to normal life conditions. 

The same authors published a study in 2021 in which they analyzed the skin microbiome of an astronaut during a one-year mission on the ISS. Samples were taken before the departure and subsequently at intervals of two or three months during the stay in space, and again finally on the return to Earth. The authors reported an increased colonization by *Malassezia restricta* and a decreased colonization by *Malassezia sympodialis* during the inflight period both in the cheek and the chest samples, thus concluding that the skin *Malassezia* microbiome in astronauts involved in half- and one-year missions is mycologically similar to that of patients with seborrheic dermatitis [41].

Changes in the composition of bacterial skin communities could also be linked to the negative impact caused by the synthetic garments used in the ISS to counteract the negative effects of microgravity on astronauts, in particular on bone and muscle density and on the dynamics of the spine. In a study published in 2017 by Stabler et al. [37], the authors evaluated changes in the skin microbiota of a European Space Agency astronaut during a 10-month pre-flight training period, an 8-day mission aboard the ISS (when the SkinSuit was worn for 6–7 h periods on two consecutive days) and a 1-month recovery period. The SkinSuit is a specific compression garment that astronauts can wear, which has a structure and a texture that counteracts the effect of microgravity and simulates the compressive forces that the human body experiences when on Earth. While confirming the predominance of Actinobacteria, Firmicutes, Proteobacteria and Bacteroidetes in the healthy skin microbiota of volunteers, the authors showed that, in the astronaut’s skin samples, the differences in phylogenetic distances between bacterial communities were particularly evident during the period aboard the ISS, but reverted to a profile similar to pre-flight composition on return. 

Another aspect of interest to researchers in this field is the analysis of not only the variations in astronauts’ skin microbiome over time, but also understanding how the skin microbiome interacts with environmental microbial flora, i.e., with the bacteria that can colonize the internal structures of the space station. In particular, Voorhies et al. [38] performed a longitudinal study on nine astronauts that were enrolled to spend from six to twelve months at the ISS. The objective of this study was to analyze the variation in the intestinal, skin, salivary and nasal microbiome, and to correlate any variations with alterations in the health status of the astronauts. Microbial samples from different locations on the astronauts’ bodies, including skin sites such as forehead and forearm skin, were collected at 10 different time points before, during and after the mission. In addition, the interplay between the ISS microbial communities and the crew’s microbiome was studied by analyzing the in-flight microbial environmental samples collected by the crew from six different surfaces and one sample from the water reservoir in the ISS. During the space travel, the authors performed a comparative analysis of the Shannon alpha diversity index and richness for each of the human body locations surveyed. In particular, analysis of the astronauts’ microbiome of the two skin sites surveyed revealed some changes in alpha diversity and richness, which became higher or lower depending on the individual, but was consistent within astronauts between forehead and forearm skin. The authors explained this differential skin microbiome response to space travel taking into account factors such as the original composition of the microbial communities of the skin, skin-specific properties such as moisture and pH, and/or the astronauts’ personal hygiene habits. The authors also noticed shifts in the microbial composition that were common across all crew members during space flight. These changes involved a significant inflight reduction in Proteobacteria, mostly, and *Betaproteobacteria*, with a concomitant increase in Firmicutes, including *Staphylococcus* spp. and *Streptococcus* spp. Since skin hypersensitivity reactions/rashes and skin infections are listed among the most common clinical episodes astronauts experience during space travel, the authors hypothesized that the occurrence of these clinical episodes may be related to the reduction in skin *Gammaproteobacteria*. Furthermore, the authors underlined that the high frequency of skin infection episodes recorded in space might be attributed to the fact that delayed cellular proliferation of the basal skin layer together with a thinning of the upper layer of the epidermis could increase the exposure of the microbial communities that reside in the deeper layers of the skin, resulting in the facilitation of the establishment of the skin infections sustained by opportunistic pathogens, such as *Staphylococcus* spp. and *Streptococcus* spp, that increased during space flight. 

According with that previously shown regarding the rapid transfer of skin-associated microbes from individuals to the areas they reside in [25], Voorhies et al. [38] described a strong interaction between the forehead and forearm skin microbiome of astronaut crew members and the ISS environments, with the surfaces of the ISS resembling the astronauts’ skin microbiomes. Interestingly, the microbial communities detected on surfaces within the ISS appeared to be transient: the composition of microbes on the ISS changed shortly after one crew member departed and a new crew member arrived, reflecting the new crew member’s skin microbiome. Nevertheless, the authors found that a small proportion of the environmental bacteria were ubiquitously present in the ISS (in particular, *Corynebacterium* spp., *Staphylococcus* spp., *Streptococcus* spp. and *Cloacibacterium* spp.). The authors presumed that these bacterial communities were likely either long-term residents of the space station or microbes that are extremely common on humans. 

There has been growing interest in recent years in the role of the environmental microbiome on the delicate balance of the complex of microorganisms that colonize the human skin. In a work published in 2020, Avila-Herrera et al. [39] studied one crew member’s microbial profile collected from body swabs of different body locations, including mouth, nose, ear, skin and saliva. The samples were collected at eight different time points pre-, during and post-flight. Microbial diversity was assessed using shotgun metagenome sequencing. In order to evaluate whether the crew member’s microbiome might influence the microbial composition of the ISS, the authors also collected environmental surface samples from eight habitable locations during two different flights. In particular, environmental surface wipes were collected from one flight by a crew member. Furthermore, after the crew member’s departure, environmental samples were collected from the next flight. The results showed that the microbial composition of the skin, nostril and ear samples collected from the crew member resembled the microbial communities yielded by the ISS surfaces more than the saliva and mouth samples did. As this study was confined to one crew member subject, the statistical significance and confidence of the results was clearly very limited. Nevertheless, this work supported what was previously shown by Voorhies et al. [38] about the microbial exchange between crew members’ skin and surfaces at the ISS, with the microbial composition of the crew member’s skin samples closely related to the ISS microbiome.

In 2021, Mahnert et al. published the results of a study on the microbiome profiles of six crew members and four representative locations (toilet bowl, kitchen floor, a desk in one of the bedrooms and a desk in the main room) within the confined environment in the 1-year Hawaii Space Exploration Analog and Simulation IV (HI-SEAS) mission [40]. In this study, the authors report a retarded longitudinal homogenization between the skin and built environment samples only after 210 days. In contrast to previous findings, the microbial diversity on the HI-SEAS surface remained rather constant, and even increased in samples from crew skin. In this study, most skin-associated microorganisms were widely traced in the habitat, were most frequently exchanged with the desk surface in the bedroom, and were more likely transferred between crew members who also had close physical interaction. In particular, samples from crew members’ skin showed significantly lower diversity than samples from the surfaces of the built environment, with the skin samples characterized by *Staphylococcus* spp., *Propionibacterium* spp., *Enterobacteriaceae*, *Enhydrobacter* and *Methanobrevibacter*, whereas the built surfaces were characterized by the presence of *Chryseobacterium* spp., *Lactobacillus* spp., *Gardnerella*, *Prevotella* spp. and *Acinetobacter* spp. The findings of this study confirmed the importance of the personal microbial cloud and of direct physical interaction among crew members in the stabilization of the microbial transfer pattern.

Morrison et al. [42] carried out a deep molecular analysis of body microbiome changes in four astronauts pre-, during, and post-flight at the ISS, to characterize the astronaut’s microbiome changes due to space flight conditions and to identify any changes that may pose health risks to the astronauts during their mission. They collected samples from the skin, nose, ear, mouth and saliva of four astronauts on consecutive space flights. For the skin samples, five different sections of the skin were sampled, including forehead, armpits, antecubital fossa and the navel region. The results reported in this study did not show the four astronauts’ skin microbiomes shifting toward a consistent “ISS-space flight” microbiome while they were on board.

Overall, these few available studies have shown that:

- During their stay in space, the variability of the astronauts’ skin microbiome tends to decrease, and then returns to pre-mission levels once they return to Earth, even if, in some cases, the response is very variable, with some astronauts showing an increase in microbial variation during their stay in space; in particular, *Gamma*- and *Betaproteobacteria* spp. abundance tends to decrease, while the occurrence of the *Malassezia* species and Firmicutes, including *Staphylococcus* spp. and *Streptococcus* spp., tends to increase.
- Some living conditions in space, in particular microgravity and the impossibility of washing the skin using traditional methods, lead to very similar alterations (increased sebum production) to those found in some skin diseases, namely skin hypersensitivity reactions and skin infections; in particular, *Malassezia* species ratios have shown that *M. restricta*, which usually colonizes the skin of patients with seborrheic dermatitis, increased during the inflight period while *Malassezia sympodialis* decreased.
- Even if the mechanisms are not entirely known, there seems to be an exchange of microorganisms between astronauts and between the confined environment and a single astronaut, with alterations in the proportion of the microorganisms that are maintained during the flight, in particular for species such as *Corynebacterium* spp., *Staphylococcus* spp., *Streptococcus* spp. and *Cloacibacterium* spp.

In future aerospace research, it will be important to conduct studies monitoring the changes in the skin microbiome of astronauts even for very long periods. However, it should be borne in mind that, from a statistical point of view, we will never have large amounts of data, given that the number of astronauts who alternate in the ISS is not large and that their daily agenda is so full of commitments and technical-scientific activities that sampling is not always easy. Moreover, the effect of life habits (in particular of personal hygiene and the type of clothing used) on the variability of the microbiome has been amply demonstrated by these studies on astronauts. Therefore, in future, it will be essential to increase research in this area, not only to benefit aerospace medicine, but also to identify primary and secondary prevention strategies for some skin diseases using cosmetic and hygiene products, along with particular types of fabric for clothing.

This review’s findings should be interpreted in light of our work’s limitations. We conducted a literature search applying an English-language filter; therefore, significant findings in other languages may have been overlooked. Moreover, only two databases (Pubmed and Scopus) were used, and a relevant search term may have been omitted, with the relevant results not retrieved as a consequence. In addition to this, the transversal nature of our review and the characteristics of the included studies preclude us from drawing causal relationships or making a quantitative comparison of the studies’ results.

## 4. Conclusions

Sixty years after the launch of the first human into space, knowledge regarding the physiological changes that the human body undergoes during dynamic flight phases and prolonged weightlessness exists, including those changes relating to skin microbiota. Recent investigations have revealed the fundamental role of microbes as guardians of man; in fact, the skin microbiota takes part in protection against infections and interacts with the immune system. At the same time, however, it has been found that alterations in the same or an expansion of some microbes compared with others can lead to the development of autoimmune diseases or other inflammatory diseases and allergic reactions, with consequent abnormal inflammatory responses and tissue damage. Given that alteration in the skin microbiota can result in skin diseases, it is important to understand the skin microbiota changes that can occur during space flight to develop the preventive measures essential to ensuring astronaut health. Among other skin microbiota changes, the increased percentage of *Malassezia* has proved to be a common characteristic due to the body-washing method necessitated by space flight. Moreover, the microbiota change has been shown to be only temporary, reverting to a profile similar to that in pre-flight once there has been a return to normal life conditions. Finally, different studies suggest a straight correlation between the skin microbiota of the astronauts and the microbial profile collected on the different surfaces of the ISS. However, the relationship between crew members and space microbial profiles, and its potential effect on crew health should be further investigated. Since the rapid return of crew and Earth-based medical interventions seem implausible during space flight, systematic and spatiotemporal measurements, with the on-board collection of microbial data, will be fundamental for future mission plans. It is important to determine whether the observed alterations in the skin microbiome during space flight pose a risk to astronauts’ health. In conclusion, effective programs, including the continued monitoring and sampling of the microbiome, are needed to prevent the development of dermatologic conditions during space flight, including viral reactivations, contact dermatitis or eczematous patches, and skin infections.

## Figures and Tables

**Table 1:** Summary of the results of the systematic review. The number of subjects involved in the study is marked with * to indicate astronauts and with ** to indicate healthy volunteers.

Reference	Number of Cases (Astronauts) * (Healthy Volunteers) **	Location	Sampling Sites	Methods of Analysis	Results
Sugita et al., 2015 [35]	16 (members of geological Antarctic research expeditions)	Antarctica	Scalp, cheeks, anterior chest, behind the ear, soles of feet	Real-time PCR assay with a Taq-Man probe	– The levels of *Malassezia* species (*M. globosa* and *M. restricta*) colonization increased during the visit to Antarctica and returned to physiological levels upon their return home.
Sugita et al., 2016 [36]	10 *	ISS	Cheek, chest	Pyrosequencing of barcoded 26S rRNA gene	– *Malassezia restricta*, *Malassezia globosa*, *Malassezia sympodialis* and *Cyberlindera jadinii* colonization increased during the stay on the ISS and decreased upon return to Earth. – Opportunistic pathogens such as *Candida albicans*, *Candida tropicalis*, *Cryptococcus albidus*, *Cryptococcus laurentii* and *Tricosporon asahii* were also detected.
Stabler et al., 2017 [37]	1 * and 5 **	ISS * Earth **	Chest, lower back, armpit, groin (healthy volunteers) Armpit, back, chest, groin, dry site on the leg (astronauts)	DNA amplification and sequencing of the hypervariable V3–V4 16S rRNA region of the bacterial genome	– Surfaces on board the ISS were initially colonized with skin-associated genera such as *Staphylococcus* spp., *Micrococcus* spp., *Bacillus* spp. and *Streptococcus* spp. – The healthy skin microbiota is rich and diverse but the majority of bacteria belong to four phyla: Actinobacteria, Firmicutes, Proteobacteria and Bacteroidetes. – Higher abundance of *Staphylococcus* spp., *Propionibacterium* spp. and *Corynebacterium* spp. in the samples from Astronaut A compared with the volunteers and a higher abundance of *Micrococcus* spp. and *Paracoccus* spp. in the volunteers.
Voorhies et al., 2019 [38]	9 *	ISS	Forehead and forearm skin	DNA amplification and sequencing of the hypervariable V4 16S rRNA region of the bacterial genome	– Significant inflight reduction in Proteobacteria, mostly *Gamma* and *Betaproteobacteria*, with a concomitant increase in Firmicutes, including *Staphylococcus* spp. and *Streptococcus* spp.
Avila-Herrera et al., 2020 [39]	1 *	ISS	Forehead, armpits, navel, forearms, back of both ears	Shotgun metagenomics sequencing	– Skin samples were dominated by *Propionibacterium acnes.* – *Propionibacterium acnes* and *Staphylococcus epidermidis* are the most prevalent in all ISS surface locations analyzed.
Manhert et al., 2021 [40]	6 *	HI-SEAS IV (Hawaii Space Exploration Analog and Simulation IV)	Front torso	DNA amplification and sequencing of the hypervariable V3–V4 16S rRNA region of the bacterial genome	– Samples from the crew’s skin showed significantly lower diversity than samples from surfaces of the built environment. – Overall, the skin samples were characterized by a high abundance of *Staphylococcus* spp., *Propionibacterium* spp., *Enterobacteriaceae*, *Enhydrobacter* and *Methanobrevibacter*, whereas the built surfaces were characterized by the presence of *Chryseobacterium* spp., *Lactobacillus* spp., *Gardnerella*, *Prevotella* spp. and *Acinetobacter* spp.
Sugita et al., 2021 [41]	1 *	ISS	Cheek, chest	Fungal D1/D2 rRNA genes NGS sequencing Quantitative PCR with a Taq-Man probe	– *Candida boidinii*, *Candida tropicalis*, *Cyberlindnera jadinii*, *Malassezia globosa*, *M. restricta*, *M. sympodialis* and *Rhodotorula mucilaginosa* represented 97.8–99.9% of sequences. – The relative abundance of *M. restricta* increased during the inflight period, along with increased colonization by *Malassezia*, whereas that of *M. sympodialis* decreased, in both the cheek and chest areas.
Morrison et al., 2021 [42]	4 *	ISS	Forehead, armpits, forearms, navel	Shotgun metagenomics sequencing	– The top three most abundant genera in the skin samples were *Propionibacterium* spp., *Corynebacterium* spp., *Staphylococcus* spp. and *Malassezia restricta.* – The results did not show the astronauts’ skin microbiomes shifting toward a consistent “space flight” microbiome while they were on board the ISS.

## Data Availability

Not applicable, no new data were created or analyzed in this study.
